# Clinical Profile and Acute-Phase Management Modalities of Pediatric Hand Burn: A Retrospective Study

**DOI:** 10.3390/ebj3010005

**Published:** 2022-01-25

**Authors:** Kayhan Gurbuz, Mete Demir

**Affiliations:** Burn Center, Department of General Surgery, Adana Faculty of Medicine, University of Health Sciences, Adana 01060, Turkey; drmetedemir@hotmail.com

**Keywords:** pediatric hand burns, prevention, functional outcomes

## Abstract

Although the hands constitute approximately 5% of the total body surface area (TBSA), the sequelae and subsequent functional outcomes following hand burns (HBs) significantly impact the quality of life for affected patients. HBs, which frequently accompany severe burns and are often neglected, deserve additional attention in the management of burns of this anatomical region, as they are responsible for a majority of postburn morbidity. In addition, many questions remain unanswered in almost every aspect of HB management. Moreover, recent articles suggest that the primary issue of optimal timing concerning skin closure for HBs, which seemed well answered, has been questioned, and even this fundamental question may require further investigation. Appropriate initial management of HBs commencing from the acute phase in children remains of great importance in optimizing functional outcomes and minimizing long-term scar formation. In this context, our primary purpose in this retrospective cohort study was to present the epidemiological characteristics of HBs in children as a whole and to discuss the incidence and mechanisms, in addition to the outcomes of superficial and deep HB acute-phase management modalities. During the 5-year study period, HBs were detected in 27% (*n* = 422) of 1580 hospitalized pediatric burn patients in the Adana Faculty of Medicine-University of Health Sciences (AFM-UHS) Burn Center. Movement and functional status of the hands were evaluated with a simple qualitative staging system adapted from the original scoring tools used by Stiefel et al., and Sheridan et al. Good, moderate, and poor scores in the study were graded as Category A, Category B, and Category C, respectively. According to the hand movement and function assessment categorization, 84% of the cases were observed as Category-A who had good/normal regular movements/functions of the hands/fingers, followed by Category-B and -C with percentages of 15 and 1, respectively, during the 5.8 ± 3.4 month follow-up period.

## 1. Introduction

Although the hands constitute approximately 5% of the TBSA [1,2], the sequelae and subsequent functional outcomes of HBs significantly impact the quality of life [3]. Since the hands show a unique form of burns bearing distinctive features of anatomical structures from other anatomical sites, including across the dorsal and plantar regions of the hands, they present more difficult management modalities than burns of other body parts. HBs are a common type of burn, albeit isolated or combined with other anatomical regions’ burns; in the literature, the stated range of incidence of HBs involved in patients with severe burns was between 35% and 89% [3,4,5,6,7,8].

Children’s discovery and recognition of their environment are realized through the tactile and sensory functions of the hands. Meanwhile, young children particularly need more support to protect themselves from environmental damage and to stay away from potential harms. Delays in help and support result in prolonged exposure of children to causative burn agents and increases hand damage. Differences begin to occur in the formation of the mechanisms of HBs according to the age groups of children. The reality of the prevalence of scald burns in children under five years old gain the characteristics comparable to the etiological aspects of adult age groups within the age range from 5 to 17 [9], and is also generally valid for HBs. Flame-related burns and electrical/chemical injuries are expected in greater frequency within this age group [9]. While scalding burns are more common on the dorsal surface of the hand [10], palmar burns are most expected in children under five years of age as contact burns are caused by hot stoves, hair straighteners and open oven doors. 

HBs, especially in children, are among the leading causes of hand injury and significantly impair hand function [11,12]. Appropriate initial management of HBs in children is of great importance in optimizing functional outcomes and minimizing long-term scar formation [11]. Proper initial management can minimize further tissue damage and improve long-term functional and psychosocial outcomes [11,13]. The most appropriate HB management modalities to preserve hand functions starting from the acute phase could be counted as follows: providing the elevation procedures of the hands; the use of topical antibacterial agents [10,14]; surgical procedures such as escharotomy/fasciotomy/debridement/skin grafting; and the use of epidermal/dermal substitutes when necessary [15]. Therefore, in the acute phase, treatment of superficial partial-thickness burns through optimal local wound care and edema control generally provides healing with epithelialization, usually within 10 to 14 days, which shortens the inflammatory phase, minimizing scar formation and loss of function [16].

Conversely, when deep partial-thickness burns are allowed to heal spontaneously, the prolonged inflammatory phase causes poor re-epithelialization and dense collagen deposition, resulting in functional impairment and increased scarring. These burns are usually treated similarly to full-thickness burns [16]. Within the scope of controlled debridement of non-vital tissues in deep partial- and full-thickness HBs, in addition to surgical excision (tangential/facial), the enzymatic debridement [17] and hydro-surgery options should also be kept in mind. While various wound closure options exist, the most common are split-thickness skin grafts (STSGs). Active/passive range of motion and edema control should be ensured with post-operative splinting methods [16], and a physical therapy program should be initiated in the acute phase of the HBs.

HBs are common in the pediatric population, and the etiology, treatment, and outcomes of HBs differ between children and adults [10]. In terms of anatomical characteristics, the thinner skin structure of children makes them more prone to full-thickness burns than adults [10,18]. 

In this context, our primary aim in this retrospective cohort study was to present the epidemiological characteristics of HBs in children as a whole and discuss the incidence and mechanisms, in addition to the outcomes of superficial and deep HB acute-phase treatment modalities.

## 2. Materials and Methods

### 2.1. Data Extraction

A retrospective cohort study was undertaken, and after obtaining ethics committee approval from AFM-UHS, demographic data and clinical variables of HBs among children aged 0 to <18 years in inpatient treatment with burns between 1 January 2015, and 31 December 2019, were extracted from the AFM-UHS electronic database.

### 2.2. Inclusion Criteria

(a) Pediatric patients aged 0–<18 years with HBs cover all burn etiologies, whether isolated or accompanied by other anatomical region burns. The hospitalized pediatric patients with HBs. (b) Patients who underwent minor amputations (fingers).

### 2.3. Exclusion Criteria

(a) Patients with superficial epidermal burns. Minimal HBs followed on an outpatient basis. (b) Patients who had undergone major amputation (below/above elbow joint) with 4th-degree HBs. Moreover, patients who had exposed tendons. (c) Patients with missing medical records.

### 2.4. Treatment Algorithm for Hand Burn Injuries

Since most HBs accompany burns of other anatomical regions, fluid replacement was applied to patients as required by keeping the urine volume 0.5/cc/h and above, as per the Parkland formula. H2 receptor blockers and low molecular weight heparin (LMWH) were administered to all patients. Edema was reduced by raising the injured hands. The wound was covered with a moist dressing that allowed active/passive hand and finger range of motion by starting the physical therapy program when the pain control improved from the initial moment of the injury.

The need for an escharotomy is often made with a clinical assessment based on history (e.g., electrical, flame-related, or scald), or physical examination (e.g., circumferentially injured, coldness to touch, resistance to passive straightening of the fingers, the tension of the hand on palpation) of the patients. In addition to the above, the indications for fasciotomy were as follows:

- Failure of escharotomies to restore perfusion
- Decrease below 90 percent in peripheral pulse oximetry
- Compartment pressure exceeding 30 mmHg
- Absence of distal perfusion in Doppler USG
- Presence of myoglobinuria

The second evaluation was performed in the burn operating room under sterile conditions and anesthesia (general/local), and the necessity of escharotomy/fasciotomy was evaluated and applied for the patients as required within 8 h to 48 h, following the initial injury. Carpal tunnels were exposed upon discovery of circulation problems in electrical HBs. Moreover, the surgery practice according to the burn-depth was determined firstly during the second evaluation, and a conservative treatment method was preferred for superficial partial-thickness burns that would recover with minimal scarring and loss of function. As conservative treatment in full-thickness burns results in poor cosmetic outcomes and functional losses, the necessary controlled surgical debridement (tangential/fascial/hydro-surgery) and necrectomy procedures involved removal of the significant necrotic burden from the hand burn area in the first 24 h up to 72 h and, in some cases, continued for up to 15 days. In cases where the depth of the burn was uncertain, the following procedures were performed after surgical cleaning of the wound: application of moist wound closure using local antibacterial agents, the use of skin substitutes, and dressing changes in 2-day periods, followed by a waiting-period between ten days and the maximum of two weeks. Superficial partial-thickness burns healed spontaneously during this time, STSGs were applied to the patients who did not heal, and debridement was completed during this period. In addition, in our clinical practice, STSG 0.2–0.3 mm thick was generally used for palmar surface HBs and finger repair as full-thickness-skin grafts (FTSGs) usually contain hair follicles and were not considered suitable for palms. In the case of 0.3 mm, thicker partial-thickness, and more limited graft use, 0.2 mm thick grafts were obtained from adjacent donor sites and transferred to the affected 0.3 mm area to reduce problems such as donor site scarring and pain [18]. Kirschner wires were used for joint stabilization only in unstable exposed joints in HBs.

### 2.5. Data Analysis and Categorization of Hand Function/Scarring

Age groups were stratified as 0–4, 5–9, 10–14, and 15–<18 age groups to evaluate the clinical outcomes of HBs treated conservatively and surgically according to the age groups. 

Post-operative scar composition was evaluated using the Vancouver Scar Scale (VSS). This scoring system involves several parameters: pliability, elasticity, vascularization, and pigmentation. Each parameter has an associated number, and the cumulative score is the total of all parameters. Lower scores indicate favorable results [10,19].

Table 1 shows the burn scar evaluation obtained by the Vancouver Scar Scale.

Hand motion/functional status was assessed by a simple qualitative staging system adopted from the original scoring tools used by Sheridan et al. [20] and Stiefel et al. [21].

- Category-A. ‘‘Good’’: regular movements/functions of the hands/fingers; VSS: 0–2 (near normal skin texture, minimal scaring)
- Category-B. “Moderate”: reasonable improvements and mild limitations of the movements/functions of the hands/fingers that do not prevent the performance of activities of the daily life; VSS: 3–8 (modest textural and pigmentationally abnormalities)
- Category-C. “Poor”: no or minimal movements/functions of the hands/fingers to perform daily activities such as eating and toileting; VSS: 9–13 (significant hypertrophic scar and scar contractions)

Table 2 shows the simple qualitative grading system for the assessment of motion/functional status of the hands.

Data were analyzed using SPSS 20.0 for Windows (Statistical Package for Social Science v20) software. Data evaluations were made using Chi-square (parametric methods) and Mann–Whitney U test (non-parametric methods). Results are expressed as mean ± standard deviation (SD) (minimum–maximum). *p* Values ≤ 0.05 were considered as significant.

## 3. Results

### 3.1. Age and Gender

Table 3 shows the demographic and clinical variables of the hand burn among hospitalized pediatric patients according to the categories of the hand function.

According to the mean of age, Category-C constituted the oldest children with the age of 15.5 years (SD = 0.7, range = 15.0–16.0), followed in descending order by Category-B and -C, with 6.3 years (SD = 5.5, range = 0.5–17.0), and 3.9 years (SD = 3.8, range = 0.5–17.0), respectively (*p* < 0.001). There was no significant difference between the categories in terms of gender (*p* = 0.375).

### 3.2. Incidence of Pediatric Hand Burns-Isolated/-with Other Anatomical Site Burns

During the 5-year study period, HBs were detected in 27% (422) of 1580 hospitalized pediatric burn patients. Since the AFM-UHS Burn Center is one of the high-volume referral centers in Turkey, only hospitalized patients were included in the study. Isolated HBs were observed in only 42 patients (10.0%), while other anatomical site burns accompanied HBs in 90.0% of the cases.

### 3.3. TBSA%, and Length of Hospital Stay

HBs evaluated as Category-C were accompanied with severe burns by a mean TBSA of 42.5% (SD = 6.4, range = 38–47), and in Category-B and C, in decreasing order with TBSA percentages of 22.6 (SD = 19.6, range = 1–85%), and 8.5 (SD = 12.0, range = 1–100%), respectively (*p* < 0.001). In addition, in terms of the mean LOS, Category-B had the longest time with 42.2 ± 44.6 days, while the shortest mean of LOS was observed in the patients in Category-A with 9.7 ± 7.2 days (*p* < 0.001) (Table 3).

### 3.4. Etiology and Place of Accidents of Hand Burns

**Figure 1:**
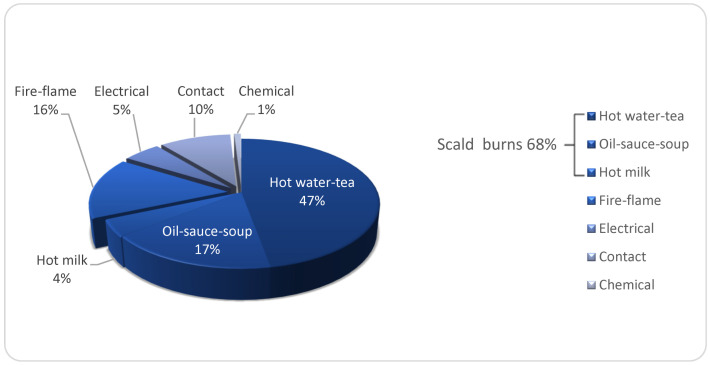
Distribution of hand burn etiology in hospitalized pediatric patients (*n* = 422).

Figure 1 shows the distribution of hand burn etiology in hospitalized pediatric patients. 

Scald burns constituted the majority of HBs constituting 68% of total HBs, followed by fire-flame-related, contact, electrical, and chemical burns with decreasing percentages of 16, 10, 5, and 1, respectively (Figure 1). While scalding burns constituted most of the etiology of dorsal HBs, representing 89% (*n* = 376) of the cases, palmar burns, which constitute 11% (*n* = 46), were caused by contact-/fire-flame-related- burns and electrical injuries. 

### 3.5. Outcomes of the Treatment Algorithm for Hand Burn Injuries

Eighty-four percent (*n* = 355) of the cases were treated conservatively within this general treatment algorithm framework. STSGs were applied to 66 hospitalized pediatric patients with HBs (16%) (*n* = 66), while groin flap repair was performed in only one patient with a high voltage (>1000 V) hand injury (Table 3). Dermal substitutes have been used in our clinic since 2017 and were used for three patients in the study group.

According to the hand movement/function assessment scoring system adapted from the originals of the studies by Stiefel et al. [21] and Sheridan et al. [20] (Table 2), 84% of the cases were observed in this study as Category-A, followed by Category-B and C with percentages of 15 and 1 (Table 3), respectively, during the mean follow up 5.8 ± 3.4 month. It should be noted that only one amputation observed in Category-A was the fourth distal phalanx minor amputation that did not interfere with the normal movement and function of the hand. Approximately 2% of the patients in Category-A required skin grafting by applying the specified treatment protocol within the burn center, while skin grafted Category-B patients (87.7%) often had outcomes that minimally interfered with their daily lives. Only two patients were identified in Category C requiring advanced reconstructive procedures and were hoped to eventually be considered in Category-B following the termination of the procedures (Table 3).

## 4. Discussion

With improvements in burn management techniques and advances in intensive care, quality of life term covers mainly burns of the hand and face, as survival rates in severe burn injuries increase. HBs, which frequently accompany severe burns and are often neglected, deserve attention in the management of burns of this anatomical region, as they are responsible for a majority of postburn morbidity [2]. In addition, many questions remain to be answered in almost every aspect of HB management. Moreover, recent articles suggest that the primary issue of optimal timing for skin closure for HBs, which seems well answered, has been questioned, and even this fundamental query may require further investigation [22]. Other phases of HB management includes the following:

- The type of the skin grafts (split-/full-thickness)
- The timing for a range of motion
- The use of splinting/Kirschner wires
- The timing of surgical treatment
- The surgical procedures to be applied for the cases in which the exposed tendons
- Moreover, the use of dermal substitutes and post-operative positioning continues to be unresolved [22].

By sharing our single-center experience, the answers to the above questions were as follows: Evaluation of the necessity of escharotomy/fasciotomy and its application to the appropriate patients were undertaken 8 h after the first injury and within 48 h at the latest; conservative treatment methods were preferred for superficial partial-thickness burns; while controlled surgical debridement (tangential/facial/hydro-surgery) and necrectomy procedures required for removal of significant necrotic load from the burn area were performed in the first 24–72 h in full-thickness burns, where this period could take up to 15 days in some patients; dermal substitutes have been used in our clinic since 2017, and they were used in three patients in the study group. Additionally, palmar surface and finger repairs were performed using 0.2–0.3 mm thick STSGs. FTSGs contain hair follicles and are not considered suitable for palms. Moreover, non-meshed STSGs are generally used to cover pediatric dorsal site HBs. It should be noted that the splints were used for the first 7 days until the sutures or clips for STSG fixation were removed. Immediately after this period, we continued the physical therapy program. After discharge, the patients were referred to the Physical Therapy and Rehabilitation Department. 

Apart from isolated, minor burns involving the hand solely, there are difficulties in adhering to strict management principles/aims/protocols in the acute phase of HBs accompanying major burns. There remains a great need to develop practical, inexpensive, sustainable dressings and treatment modalities that allow physical therapy to provide an increase in range of motion from the earliest moments of injury [2]. The basic principle of HB management is based on the depth of the burn. Superficial partial-thickness hand burns heal spontaneously within 10 days to 2 weeks, and in addition to wound care, physical therapy comes to the fore to preserve the range of motion and thus hand function. However, treating deep partial- and full-thickness burns is better treated with surgical excision and grafting [22]. In this respect, it is worth noting that many studies have been conducted on the management of deep partial-thickness and full-thickness HBs [3,23,24,25,26,27]. The STSGs remains our surgical method of preference to treat burns on the dorsal surface of the hand. Prasetyono et al. stated that there was no agreement among the included studies in their systematic review regarding which of the FTSGs and STSGs was better in covering pediatric volar side HBs [28]. Although FTSGs are recommended with flap use in hand palmar burn injuries, the probability of graft failure in the presence of infection and edema is higher than STSGs. The donor area is limited, and its use in severe burns is impractical and unrealistic. 

In this study, HBs accompanying burns of other anatomical regions were found in 27.0% (422/1580) of the cases and were lower than studies reporting the incidence range of HBs as 35–89% in previous studies [3,4,5,6,7,8]. 

As mentioned above, the management of HBs in burn centers and the optimal assessment of treatment outcomes remains challenging due to the lack of validated assessment tools related to the comprehensive data collected [22]. The unique anatomical features of the hand remain one of the main topics in the analysis of the range of motion at each joint, hand strength and sensation, and overall functional outcomes [22]. Moreover, some of the outcome tools described on HBs have not been validated for burn injuries, and uncertainty remains whether they respond to hand function improvement over time and effectively identify burn patients’ problems [22]. The postoperative scar composition was evaluated in the current study using VSS [10,19]. Additionally, motion/functional status of the hands were assessed by a simple qualitative staging system adopted from the original scoring tools used by Sheridan et al., [20] and Stiefel et al. [21]. In the meantime, similar to the findings in the study by Sheridan et al., the success rate of functional gain was 97% for superficial partial-thickness burns and 81% for deep partial- and full-thickness burns [23]. In the current study, according to the hand movement/function assessment scoring, 84% of the cases were observed as Category-A who showed good/normal regular movements/functions of the hands/fingers, followed by Category-B and C with percentages of 15 and 1, respectively, during the 5.8 ± 3.4 month of follow-up period. 

### 4.1. Limitations of the Study

The fact that the study was conducted as a retrospective file search in the medical records of the Burn Center of AFM-UHS presented a limitation. Consideration of the records of only inpatients, exclusion of outpatient HBs, which constitute approximately 90% of the patient population, can be counted among the limitations of this study.

### 4.2. Conclusions

Optimal HB management modalities starting from the acute phase include providing elevation procedures, topical antibacterial agents, surgical procedures such as escharotomy/fasciotomy/debridement/skin grafting, the use of epidermal/dermal substitutes when necessary are of great importance in optimizing functional outcomes and minimizing long-term scar formation. In the study, only about 2% of patients in Category-A needed skin grafts, while this rate increased to approximately 88% in Category-B. Among Category-B patients who underwent skin grafting, no results were observed that seriously interfered with the performance of activities of daily living. In addition to skin grafting, we believe these stated “acute-phase management modalities” further contributed to the noted outcomes.

## Figures and Tables

**Table 1:** Burn scar evaluation obtained by the Vancouver Scar Scale (VSS) [19].

Parameter	Finding	Score
Pigmentation	Normal	0
	Hypopigmentation	1
	Hyperpigmentation	2
Vascularity	Normal	0
	Pink	1
	Red	2
	Purple	3
Elasticity	Normal	0
	Flexible	1
	Semi-flexible	2
	Unflexible	3
	Band	4
	Contracture	5
Height	Flat	0
	0–<2 mm	1
	≥2–<5 mm	2
	≥5 mm	3
Total score		13

**Table 2:** The simple qualitative grading system for the assessment of hands’ motion/functional status.

Grade	Characteristics
Category A (=Good)	Regular movements/functions of the hands/fingers; VSS: 0–2 (near normal skin texture, minimal scaring)
Category B (=Moderate)	Reasonable improvements and mild limitations of the movements/functions of the hands/fingers that do not prevent the performance of activities of the daily life; VSS: 3–8 (modest textural and pigmentationally abnormalities)
Category C (=Poor)	No or minimal movements/functions of the hands/fingers to perform daily activities such as eating and toileting; VSS: 9–13 (significant hypertrophic scar and scar contractions)

**Table 3:** Demographic and clinical variables of the hand burn among hospitalized pediatric patients according to the categories of the hand function.

	Category A	Category B	Category C	
	*n* = 355	*n* = 65	*n* = 2	
Variables	Mean ± SD ^a^	Mean ± SD ^a^	Mean ± SD ^a^	*p*-Value
Age (year)	3.9 ± 3.8 (0.5–17.0)	6.3 ± 5.5 (0.5–17.0)	15.5 ± 0.7 (15.0–16.0)	<0.001
TBSA (%)	8.5 ± 12.0 (1–100)	22.6 ± 19.6 (1–85)	42.5.0 ± 6.4 (38–47)	<0.001
LOS (day)	9.7 ± 7.2 (1–65)	47.2 ± 44.6 (4–258)	20.0 ± 4.2 (17–23)	<0.001
**Variables**	***n* (%)**	***n* (%)**	***n* (%)**	
**Gender**				0.375
Male	208 (58.6)	35 (53.8)	2 (100.0)	
Female	147 (41.4)	30 (46.2)	0 (0.0)	
**Age-group**				<0.001
0–4	283 (79.7)	35 (53.8)	0 (0.0)	
9–5	29 (8.2)	13 (20.0)	0 (0.0)	
14–10	34 (9.6)	7 (10.8)	0 (0.0)	
15–<18	9 (2.5)	10 (15)	2 (100.0)	
**Burn Depth**				<0.001
Superficial partial thickness	331 (93.2)	0 (0.0)	0 (0.0)	
Deep partial thickness	24 (6.8)	20 (30.8)	0 (0.0)	
Full thickness	0 (0.0)	45 (69.2)	2 (100.0)	
**Need for surgery**				<0.001
Escharotomy/fasciotomy	6 (1.7)	15 (23.1)	2 (100.0)	
Skin graft (split-/full-thickness)	8 (2.3)	57 (87.7)	2 (100.0)	
Amputation	1 (0.3)	6 (10.8)	0 (0.0)	

^a^ mean ± standard deviation (minimum-maximum); TBSA, total body surface area; LOS, length of hospital stay; STSG, split-thickness skin graft; VSS, Vancouver Scar Scale.

## Data Availability

All data are contained within the article.
